# Neuropathic pain in hereditary coproporphyria

**DOI:** 10.12669/pjms.292.3202

**Published:** 2013-04

**Authors:** Guan-Liang Chen, Deng-Ho Yang, Jeng-Yuau Wu, Chia-Wen Kuo, Wen-Hsiu Hsu

**Affiliations:** 1 Guan-Liang Chen, Department of Internal Medicine, Taichung Armed Forces General Hospital, Taichung, Taiwan, Republic of China.; 2 Deng-Ho Yang, Institute of Medicine, Chung Shan Medical University, Taichung, Taiwan, Republic of China. Department of Internal Medicine, Taichung Armed Forces General Hospital, Taichung, Taiwan, Republic of China.; 3 Jeng-Yuan Wu, Division of Thoracic Surgery, Buddhist Tzu Chi General Hospital, Taichung Branch and College of Medicine, Tzu Chi University, Hualien, Taiwan, Republic of China.; 4 Chia-Wen Kuo, Department of Internal Medicine, Taichung Armed Forces General Hospital, Taichung, Taiwan, Republic of China.; 5 Wen-Hsiu Hsu, Department of Internal Medicine, Taichung Armed Forces General Hospital, Taichung, Taiwan, Republic of China.

**Keywords:** Hereditary coproporphyria, Polyneuropathy, Neuropathic pain, Photosensitivity

## Abstract

Acute porphyrias are rare diseases with varying incidences worldwide. These diseases are disorders of heme biosynthesis characterized by acute attacks of neurological symptoms. Acute porphyria should be considered in patients with unexplained abdominal pain or neurological damage. Clinical manifestations of acute porphyria are nonspecific and are associated with multiple organ systems. This report examines a rare case of an uncommon type of acute porphyria in a patient with an initial presentation of abdominal pain and progressive polyneuropathy.

## INTRODUCTION

Acute porphyrias are rare disorders caused by a partial defect in heme biosynthesis. They are characterized by acute attacks of neurological symptoms, whereas cutaneous porphyrias are characterized by skin manifestations. The incidence rate of acute porphyria is 0.3 per 100,000 in Europe^1^, but only few cases have been reported in Taiwan. This disorder may be inherited with variable penetrance or may be acquired because of liver dysfunction. Acute attacks are rare before puberty and after menopause, with a peak occurrence in the third decade. Analyses of genetic and enzymatic features reveal that acute porphyrias are of two types: acute intermittent porphyria (AIP) and 5-aminolevulinic acid dehydratase porphyria (ADP). There are five types of cutaneous porphyrias: congenital erythropoietic porphyria, porphyria cutanea tarda (PCT), hepatoerythropoietic porphyria, erythropoietic protoporphyria, and X-linked dominant erythropoietic protoporphyria.^2^ Particularly, variegate porphyria (VP) and hereditary coproporphyria (HCP) may present as acute or cutaneous porphyria. Each type of porphyria is associated with a different partial defect in enzymes in the heme biosynthesis pathway. Clinical manifestations of acute porphyria are almost nonspecific, and the symptoms are often associated with multiple organ systems, such as abdominal pain, vomiting, constipation, weakness, hypertension, tachycardia, sensory loss, and respiratory failure.^3^^,^^4^ Acute porphyrias should always be considered in patients with unexplained or refractory abdominal pain or neurological damage. Here, we report a rare case of an uncommon type of acute porphyria, i.e., HCP, presenting with recurrent abdominal pain and progressive polyneuropathy.

## CASE REPORT

A 46-year-old Chinese woman from a low-income family, living with her mother and two sons in the suburb of Taichung City, had a history of recurrent abdominal pain. Within two years, she visited the gastroenterology clinic many times with the same complaint. There was no remarkable family history. Likewise, results of her abdominal ultrasound, endoscopy, and colonoscopy were within normal limits. The patient reported intermittent episodes of anxiety and general weakness that became progressively worse over time. Blood tests from previous visits revealed slightly abnormal liver and renal function. Chronic hepatitis C infection was diagnosed on the basis of these findings. One month before this admission, the patient presented with acute abdominal pain and progressive bilateral weakness and pain in the limbs. She also experienced significant muscle atrophy and decreased strength. During admission, the patient reported moderate, sharp pain (pain score: 5) in extremities, back, chest, and neck. She also experienced episodes of sinus tachycardia and acute urinary retention, suggesting autonomic neuropathy. The patient also reported an unusual “floating sensation” in the trunk and extremities, which was suggestive of a proprioceptive defect. In addition to anxiety, minor behavioral changes, including confusion and signs of depression, were observed. Results of a nerve conduction potential study revealed motor-sensory polyneuropathy. A motor conduction study revealed a reduced compound muscle action potential amplitude, slowed nerve conduction velocity, delayed distal latency, and prolonged F latency over the median, ulnar, tibial, and peroneal nerves.

**Fig.1:**
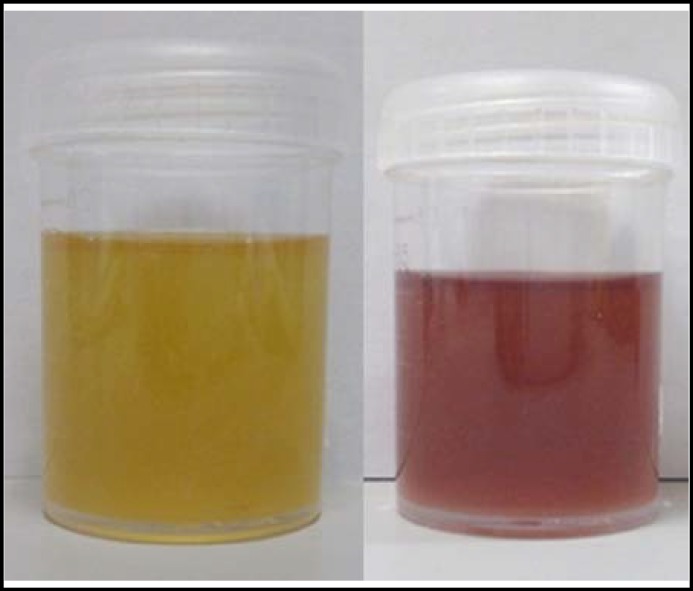
Change in urine color before and after sun exposure Left figure is urine of the first day. Right figure is urine after sun exposure for 3 days. Urine color changed to “port wine” color after sun exposure. This color change is due to increased concentrations of porphyrin intermediates in the urine, indicating an abnormality in production and a partial block within the enzymatic porphyrin chain with metabolite formation. The urine color usually becomes darker with acute illness, even dark reddish or brown after sun exposure.

 A sensory conduction study of the median, ulnar, and sural nerves revealed reduced sensory nerve action potential amplitude and delayed onset latency. Computed tomography of the brain was normal, and examination of cerebrospinal fluid showed no remarkable findings. Initial differential diagnoses included acute porphyria, lead toxicity, and vasculitis. Serum lead levels and autoimmune titers were normal, thus ruling out lead poisoning or vasculitis. A simple urine test was performed in which the urine sample of the patient was exposed to sunlight for 3 days. The urine changed from a normal yellow to port wine color after 3 days because of the accumulation of increased concentrations of porphyrin intermediates (Fig.1). *Hoesch* and *Watson*–*Schwartz urine tests were also performed. These tests revealed* increased levels of coproporhyrin and porphyrin precursors such as porphobilinogen (PBG) and aminolevulinic acid. A diagnosis of acute porphyria was thereby confirmed. Furthermore, PBG deaminase activity was within the normal range, suggesting a specific diagnosis of HCP, since patients with AIP have low PBG deaminase activity, those with ADP have normal PBG levels, and those with VP often exhibit cutaneous manifestations. The patient was initially treated with intravenous glucose 200 mg/day and cimetidine 300 mg twice daily. Abdominal pain and polyneuropathy showed mild improvement after initiating therapy. Soon after, she received intravenous hemin therapy, and the duration and severity of her abdominal pain decreased. The patient then continued to receive regular rehabilitation therapy for motor and speech deficits. After two months, the patient had no recurrence of symptoms, and her muscle strength improved significantly. She was instructed to avoid predisposing factors such as sunlight and nonsteroidal anti-inflammatory drugs.

## DISCUSSION

Acute porphyria may manifest in a number of forms including AIP, ADP, VP, and HCP. HCP is the third most common type of acute porphyria after AIP and VP. The disease most commonly occurs in Europe and North America. A prevalence of two per million has been estimated in Denmark^5^, but only a few cases have been reported in Taiwan.^6^ Most patients may have no symptoms. The common symptoms of HCP are unexplained abdominal pain, nausea, vomiting, constipation, neurovisceral symptoms, and erosive photodermatosis. Neurovisceral manifestations in HCP are not distinguishable from those observed in AIP and VP. Cutaneous manifestations in HCP are also similar to those in PCT and VP; however, they are relatively uncommon in HCP. In the present case, patient’s major manifestations were unexplained abdominal pain, peripheral neuropathy, and mental disturbance. No cutaneous manifestations were observed in this patient. Because of the rare prevalence and nonspecific manifestations of this disease, HCP diagnosis is often difficult. The differential diagnosis of acute porphyria should always be considered in patients with unexplained abdominal pain occurring after adequate survey or with presentation of polyneuropathy. In addition to the nonspecific symptoms and signs of acute porphyrias, a specific finding of HCP is darkening of urine that coincides with symptom exacerbation as noted in the present case.^7^^,^^8^ Furthermore, urine becomes dark reddish or brown in color after sun exposure. Although PBG present in the patient’s urine, it metabolizes to corproporphyrins after sun exposure, which leads to change in urine color. In contrast, alkaptonuria is a deficiency of homogentisic acid oxidase because of which urine turns black after air exposure. 

It is noteworthy that the patient in this case also had hepatitis C. Although PCT is highly associated with hepatitis C, patients with this form of porphyria only exhibit cutaneous manifestations. There is no evidence between hepatitis C and HCP. Although family history is not remarkable in this case, variable penetrance is often considered in patients with HCP, especially in fertile women in whom female hormones are known to be a predisposing factor. 

Therapeutic options for treating patients with HCP include the following: (1) hemin 3–4 mg/kg/day; (2) varbohydrate loading 300–500 mg/day; (3) symptomatic treatment for pain relief or psychological abnormality; (4) avoidance of factors that trigger exacerbations, such as sunlight, stress, infection, alcohol, barbiturate, sulfonamide, valproic acid, estrogen, and ergot. Hemin therapy exerts a negative feedback in the heme pathway and therefore should be administered early during acute attacks, and intravenous glucose is an alternative if hemin is unavailable.^9^ Prevention relies on avoiding exacerbating factors and administration of weekly hemin infusion. Genetic counseling is also suggested for family members. Complications of HCP include polyneuropathy, seizure, psychological abnormality, hypertension, chronic kidney disease, elevated serum transaminases, and hepatocellular carcinoma. Fatal complications, such as respiratory failure, may occur in an acute attack. Mortality in patients with acute porphyria is 3-fold higher than that in the general population. 

In conclusion, acute porphyria is often missed in initial differential diagnosis, resulting in inappropriate therapy and weeks of follow-up in gastrointestinal or psychological clinics. This is fortunate because screening tests are simple and accessible. Otherwise, inappropriate therapy for this disorder may lead to irreversible complications.

## References

[1] Elder GH (1997). Hepatic porphyrias in children. J Inherit Metab Dis.

[2] Puy H, Gouya L, Deybach JC (2010). Porphyrias. Lancet.

[3] Asselbergs FW, Kremer Hovinga TK, Bouwsma C, van Ingen (2009). J Am J Crit Care.

[4] Anderson KE, Bloomer JR, Bonkovsky HL (2005). Recommendations for the diagnosis and treatment of the acute porphyrias. Ann Intern Med.

[5] With TK (1983). Hereditary coproporphyria and variegate porphyria in Denmark. Dan Med Bull.

[6] Tu JB, Blackwell RQ, Feng YS (1971). Clinical and biochemical studies of hereditary hepatic porphyria in Chinese subjects in Taiwan. Metabolism.

[7] Soundravally R, Goswami K, Nandeesha H, Koner BC, Sethuraman KR (2008). Acute intermittent porphyria: diagnosis per chance. Indian J Pathol Microbiol.

[8] Bari AU (2007). Congenital erythropoietic porphyria in three siblings. Indian J Dermatol Venereol Leprol.

[9] Anderson KE, Bloomer JR, Bonkovsky HL (2005). Recommendations for the diagnosis and treatment of the acute porphyrias. Ann Intern Med.

